# PRETTY GOOD PRACTICES: GERIATRICS WORKFORCE ENHANCEMENT PROGRAMS AND LIFELONG DISABILITIES

**DOI:** 10.1093/geroni/igz038.2754

**Published:** 2019-11-08

**Authors:** Catherine Taylor, Kelly Munly

**Affiliations:** 1 Rhode Island Geriatric Education Center, University of Rhode Island, Kingston, Rhode Island, United States; 2 Penn State Altoona, Altoona, Pennsylvania, United States

## Abstract

Growing old with lifelong disabilities is a recent reality that is catching healthcare providers unprepared. While there’s little extant federal or state public policy on aging with lifelong disabilities, and aging, disability, and healthcare systems lack a history of intersystem collaboration, Geriatrics Workforce Enhancement Programs (GWEPs) can lead the way in developing curricula, training, policy, and standards to respond. The GWEPs can intervene to create meaningful intersystem knowledge and skills and better prepare providers. Two GWEPs are filling the best practices void, operationalizing de facto public policy and “pretty good” practices to improve care for individuals with lifelong disabilities. In metro Richmond, VA, the GWEP at the Virginia Geriatric Education Center (VGEC) has built on the successful Area Planning and Services Committee on Lifelong Disabilities (APSC) intersystem partnership to provide this expertise. In Rhode Island, the RI Geriatric Education Center (RIGEC) has aligned its GWEP Alzheimer’s disease supplemental funding with other federally funded programs to build dementia capability into the systems that support adults with intellectual or developmental disabilities (I/DD). RIGEC incorporated expertise previously gained through the University of Rhode Island’s CMS-funded LivingRIte Innovation, which established pilot centers to support individuals with I/DD living with dementia and other chronic conditions, through novel person-centered approaches. This symposium examines how the two GWEPs expanded upon a foundation of previous efforts to serve older adults with lifelong disabilities, the methods by which they built and fostered effective networks, the resulting system improvements, and suggested strategies to move from “pretty good” to best practices.

